# Patients’ perspectives on pulmonary rehabilitation for pulmonary hypertension in high-altitude areas: a mixed-methods study

**DOI:** 10.3389/fpubh.2026.1869309

**Published:** 2026-08-17

**Authors:** Wenrui Li, Xiangqun Ke, Jinpeng Ma, Rui Jin, Haiyu Zhao, Yu Zheng, Xiaozhou Wang, Yaping Zeng, Mingwei Chen, Tinghua Hu

**Affiliations:** 1 Department of Respiratory and Critical Care Medicine, The First Affiliated Hospital of Xi’an Jiaotong University, Xi’an, China; 2 Department of Respiratory and Critical Care Medicine, Qinghai Cardio-Cerebrovascular Specialty Hospital, Research Center of High Altitude Medicine, Xining, China; 3 Department of Hypertension, Qinghai Cardio-Cerebrovascular Specialty Hospital, Research Center of High Altitude Medicine, Xining, China; 4 Department of Coronary Heart Disease, Qinghai Cardio-Cerebrovascular Specialty Hospital, Research Center of High Altitude Medicine, Xining, China; 5 The Coronary Heart Disease Center, Beijing Anzhen Hospital, Capital Medical University, Beijing, China

**Keywords:** high-altitude, mixed-methods study, pulmonary hypertension, pulmonary rehabilitation, rehabilitation barriers, support needs

## Abstract

**Background:**

Chronic hypoxia at high altitudes worsens pulmonary hypertension (PH), leading to earlier symptom onset, more frequent exacerbations, and diminished quality of life. While pulmonary rehabilitation (PR) is known to enhance exercise capacity and ease breathlessness, implementing it in high-altitude settings presents a range of multifaceted challenges. Research capturing patients’ firsthand experiences with PR in these settings is largely absent. This study explored how PH patients in high-altitude areas currently engage with PR, what barriers they encounter, and what support they need to inform better rehabilitation strategies.

**Methods:**

A mixed-methods design was utilized, combining a questionnaire survey of 206 PH patients with semi-structured interviews of 11 patients from a tertiary hospital in Xining, Qinghai Province. Quantitative data were analyzed using descriptive statistics, whereas qualitative data were analyzed using thematic analysis.

**Results:**

Quantitative findings showed that 53.88% of patients reported engaging in breathing exercises or physical training. Among the 111 participants who reported engaging in PR exercises, 90.09% perceived improvement in dyspnea and 87.39% perceived improvement in fatigue. The leading obstacles were high-altitude environmental constraints (54.85%), lack of professional guidance (41.75%), and technical complexity such as mastering breathing training techniques (35.92%). Patients most wanted telemedicine consultations (82.04%), disease education manuals (65.05%), and family nursing skills training (61.65%). Qualitative interviews uncovered three main themes, namely current PR practices and perceived symptom changes, barriers to PR implementation, and support needs related to PR, with eight subthemes. The qualitative findings were broadly consistent with and complementary to the quantitative results: although participants reported symptom improvement, multiple barriers impede consistent participation. Patients called for structured discharge planning, community-led rehabilitation, and remote follow-up options.

**Conclusion:**

Participants reported perceived improvements in dyspnea and fatigue after engaging in PR activities; however, PR implementation remained constrained by environmental constraints, insufficient professional guidance, and limited patient awareness. Patients expressed strong preferences for telemedicine, educational materials, community-based programs, and improved discharge planning. These findings highlight the need to strengthen standardized inpatient PR education and structured discharge planning to support continuity of rehabilitation from hospital to home and may inform the development and future evaluation of context-specific PR strategies for patients with PH living at high altitude.

## 1 Introduction

Pulmonary hypertension (PH) is a progressive cardiopulmonary vascular disorder characterized by elevated pulmonary vascular resistance and pulmonary arterial pressure, resulting from a variety of etiologies, including chronic lung disease and long-term hypoxic exposure (1, 2). In high-altitude areas, long-term exposure to extreme environments such as hypoxia, low atmospheric pressure, cold, and strong ultraviolet radiation significantly increases the risk of PH, accelerates disease progression, and seriously affects patients’ quality of life and life expectancy (3). Epidemiological studies have reported that the prevalence of high-altitude pulmonary hypertension (HAPH) ranges from approximately 5 to 18% among populations living above 3,200 m, although considerable regional variation exists (4). Compared with patients living at low altitude, those residing at high altitude tend to experience earlier symptom onset, more frequent clinical deterioration, and greater impairment in daily functioning (5).

Pulmonary rehabilitation (PR) is a comprehensive, multidisciplinary intervention for patients with chronic respiratory diseases that integrates individualized exercise training with education, breathing training, psychological support, nutritional counseling, and self-management strategies to improve physical function, symptom control, and quality of life (6, 7). Current evidence suggests that PR may improve exercise tolerance, alleviate dyspnea, enhance psychological well-being, and promote long-term disease self-management in patients with PH (1, 8). Despite these established benefits, implementing PR in high-altitude areas remains challenging. The hypoxic environment not only limits patients’ exercise capacity but also increases the risk of exercise-related adverse events (9). Additionally, geographical constraints hinder patients in remote areas from accessing timely and effective rehabilitation support (10, 11). Moreover, to our knowledge, no evidence-based international guideline currently provides specific recommendations for PR in patients with PH living at high altitude. Existing recommendations are largely derived from studies conducted in low-altitude settings and provide limited guidance for adapting rehabilitation strategies to chronic hypobaric hypoxia and the unique environmental, healthcare, and cultural characteristics of high-altitude regions. A recent study from our team examined these same issues from the perspective of medical staff, identifying low adoption rates and a lack of high-altitude-specific guidelines as major systemic barriers (12). Nevertheless, the patient voice—their direct experiences, barriers, and needs—has remained largely unexplored. As the direct recipients of PR, patients’ lived experiences are essential for designing programs that actually work.

Qinghai Province is located in the northeastern part of the Qinghai-Tibet Plateau, with an altitude ranging from 1,647 meters to 6,851 meters (13). This study investigated PH patients from various areas of Qinghai admitted to the respiratory department of a tertiary hospital in Xining City, the capital of Qinghai Province. The study aimed to comprehensively understand the current practices, barriers, and support needs of patients in this region during PR and propose targeted optimization pathways based on patient needs. The findings may provide a basis for improving rehabilitation management for patients with PH living in high-altitude areas.

## 2 Methods

### 2.1 Study design

This study employed a convergent parallel mixed-methods design, in which quantitative and qualitative data were collected during the same study period, analyzed separately, and integrated during interpretation. Ethical clearance was obtained from the institutional review board (No. QXYYLL-2024-87).

### 2.2 Study participants

Participants were patients with PH who were recruited during hospitalization in the Department of Respiratory and Critical Care Medicine of a tertiary hospital in Xining. Inclusion criteria were: (1) Participants had a documented diagnosis of PH established by the treating physician and recorded in the hospital medical records according to routine clinical practice; (2) residence at high altitude for at least 1 year; (3) age 18 years or older; (4) at least two hospitalizations for PH (including the current hospitalization); and (5) provision of informed consent. Exclusion criteria were: (1) cognitive impairment, communication difficulties, or limited expressive ability; (2) other severe organic diseases such as end-stage cancer or advanced liver or kidney failure.

The minimum sample size for the quantitative survey was estimated using a participant-to-item ratio of 5:1, referring to a commonly adopted empirical recommendation originally proposed in questionnaire and psychometric research (14). Based on the 27 questionnaire items and allowing for an additional 10% to compensate for incomplete or invalid questionnaires, the minimum required sample size was 149 participants. Purposive sampling was used to recruit participants from those who had completed the questionnaire. Participants who were willing to participate in face-to-face interviews and were able to communicate effectively were invited. Recruitment continued until thematic saturation was achieved, defined as no new themes emerging from two consecutive interviews. All interview participants were clinically stable and able to communicate effectively at the time of the interviews.

### 2.3 Research tools

#### 2.3.1 Quantitative research tools

Based on the research purpose and a review of relevant literature, a self-designed non-scale questionnaire for PH patients was developed. After completing the initial draft, five experts in the fields of high-altitude clinical medicine and nursing were invited to review the questions, focusing on evaluating their validity, relevance, and clarity. Researchers optimized the question order and options based on expert feedback to form a pre-test version. Subsequently, a pre-test was conducted with 10 PH patients, with an average completion time of 8.3 min. No difficulties were encountered during the questionnaire completion, indicating its good applicability. The final questionnaire comprised 27 items across four domains: (1) participant characteristics; (2) current status of PR; (3) rehabilitation barriers and support needs; (4) additional suggestions or opinions from patients regarding PR services. Details are provided in the Supplementary material questionnaire.

#### 2.3.2 Qualitative research tools

Combined with the study objectives and a review of the relevant literature, and following consultation with experts in high-altitude medicine and nursing, a preliminary interview guide was developed. Two PH patients participated in pilot interviews to assess the clarity, relevance, and comprehensiveness of the interview questions. Based on the pilot interviews, the interview guide was refined and finalized. The final semi-structured interview guide comprised seven broad domains: current PR practices, perceived symptom changes, barriers to rehabilitation, support needs, preferred rehabilitation settings, the use of digital technologies to support rehabilitation, and suggestions for improving PR services (Supplementary Table S1).

### 2.4 Data collection process

#### 2.4.1 Quantitative data collection

Data were collected from April to July 2025. Eligible patients were recruited using a convenience sampling strategy during the discharge preparation process. The questionnaire was administered online via the “Wenjuanxing” platform. Ward nurses assisted eligible patients in accessing the survey and instructed them to answer the questions based on their overall PR experiences, including previous hospitalizations and home-based rehabilitation. Electronic informed consent was obtained before questionnaire completion. Family caregivers were permitted to assist eligible patients who were unable to complete the questionnaire independently because of illiteracy or lack of smartphone/WeChat access. Upon completion, participants were eligible to receive a small random monetary reward (RMB 1–3), which was automatically distributed through the “Wenjuanxing” platform regardless of their responses. The survey was anonymous, and neither ward nurses nor treating healthcare professionals had access to participants’ responses.

#### 2.4.2 Qualitative data collection

Written informed consent was obtained from all interview participants before the interviews were conducted. All interviews were conducted while participants were clinically stable and preparing for hospital discharge. Interviews were conducted in a quiet conference room of the hospital or in the patient’s ward (with consent), with each interview lasting 15–30 min. The interviews were conducted by a researcher (Wenrui Li) who had received systematic training in qualitative research, with another team member (Yu Zheng) observing and recording interview details. All interviews were recorded with the patient’s consent and transcribed into text within 24 h, with personal identifiers removed to protect privacy.

### 2.5 Data analysis methods

#### 2.5.1 Quantitative data analysis

Data were exported from the “Wenjuanxing” platform, organized in Excel format, and imported into SPSS 26.0 statistical software for analysis. Count data were expressed as rates or percentages. Origin 2021 software was used to generate figures.

#### 2.5.2 Qualitative data analysis

Braun and Clarke’s six-step thematic analysis method was adopted (15). Coding and theme development were independently conducted by the lead interviewer and one research team member. Cross-verification was performed to reduce subjective bias, and the final thematic framework was determined through collective discussion within the research team. NVivo 15 software was used to assist in the analysis process.

#### 2.5.3 Mixed-methods data integration

Quantitative and qualitative data were analyzed independently. Quantitative findings were used to characterize patients’ PR practices, barriers, and support needs, whereas qualitative findings provided contextual insights into participants’ experiences. The two sets of findings were then integrated through comparison to identify areas of convergence, complementarity, and divergence, thereby providing a more comprehensive understanding of patients’ PR practices, barriers, and support needs.

## 3 Results

### 3.1 Quantitative research results

#### 3.1.1 Participant characteristics

Although the estimated minimum sample size was 149 participants, recruitment continued throughout the predefined study period, and all eligible patients who were willing to participate were invited to complete the questionnaire. Consequently, a total of 206 valid questionnaires were collected and included in the final analysis. The majority were male (56.31%), aged 61 or older (74.76%), and of Han ethnicity (70.39%). Manual laborers formed the largest occupational group (34.47%). Most lived at elevations of 2,000 m or higher (88.83%) and had resided in their current home for over 30 years (77.18%). In the prior year, 76.70% had been hospitalized at least once. Additionally, 76.70% owned home oxygen equipment, 33.50% reported a one-way travel time of more than 1 h to the nearest medical facility, and 23.30% said treatment costs severely affected their daily lives. Participant characteristics are presented in Table 1.

**Table 1:** Participant characteristics (*n* = 206).

Item	Frequency (*n*)	Percentage (%)	Cumulative percentage (%)
Sex			
Male	116	56.31	56.31
Female	90	43.69	100.00
Age group			
41–50 years	11	5.34	5.34
51–60 years	41	19.90	25.24
≥61 years	154	74.76	100.00
Ethnicity			
Han	145	70.39	70.39
Tibetan	29	14.08	84.47
Hui	22	10.68	95.15
Tu	6	2.91	98.06
Manchu	1	0.49	98.54
Mongol	1	0.49	99.03
Tujia	1	0.49	99.51
Salar	1	0.49	100.00
Employment status			
Manual laborer	71	34.47	34.47
Casual/part-time worker	55	26.70	61.17
Retired	51	24.76	85.92
Homemaker	15	7.28	93.20
Professional/manager	14	6.80	100.00
Altitude of residence			
<2,000 m ASL	23	11.17	11.17
2,000–2,500 m ASL	112	54.37	65.53
2,501–3,000 m ASL	54	26.22	91.75
3,001–3,500 m ASL	8	3.88	95.63
3,501–4,000 m ASL	8	3.88	99.51
4,001–4,500 m ASL	1	0.49	100.00
Duration of residence			
1–5 years	10	4.85	4.85
6–10 years	5	2.43	7.28
11–20 years	21	10.19	17.48
21–30 years	11	5.34	22.82
≥31 years	159	77.18	100.00
Smoking			
Never smoked	112	54.37	54.37
Current smoker	75	36.41	90.78
Former smoker	19	9.22	100.00
Alcohol			
Never drank	118	57.28	57.28
Current drinker	74	35.92	93.20
Former drinker	14	6.80	100.00
Number of hospitalizations in the past year (excluding the current one)			
0 times	48	23.30	23.30
1 time	60	29.13	52.43
2 times	43	20.87	73.30
3 times	25	12.14	85.44
4 times	9	4.37	89.81
≥5 times	21	10.19	100.00
Presence of oxygen therapy equipment at home			
Yes	158	76.70	76.70
No	48	23.30	100.00
Travel time to the nearest medical facility (one-way)			
<30 min	70	33.98	33.98
30–60 min	67	32.52	66.50
> 1 h to ≤ 2 h	34	16.50	83.01
>2 h	35	16.99	100.00
Impact of treatment costs on daily life and treatment			
No impact	44	21.36	21.36
Some impact	114	55.34	76.70
Severe impact	48	23.30	100.00
Number of co-residing family members			
None	8	3.88	3.88
1 person	36	17.48	21.36
2 persons	33	16.02	37.38
3 persons	42	20.39	57.77
≥4 persons	87	42.23	100.00
Primary caregiver			
Children	115	55.83	55.83
Spouse	64	31.07	86.90
Other	27	13.11	100.00

#### 3.1.2 Current status of PR

**Figure 1:**
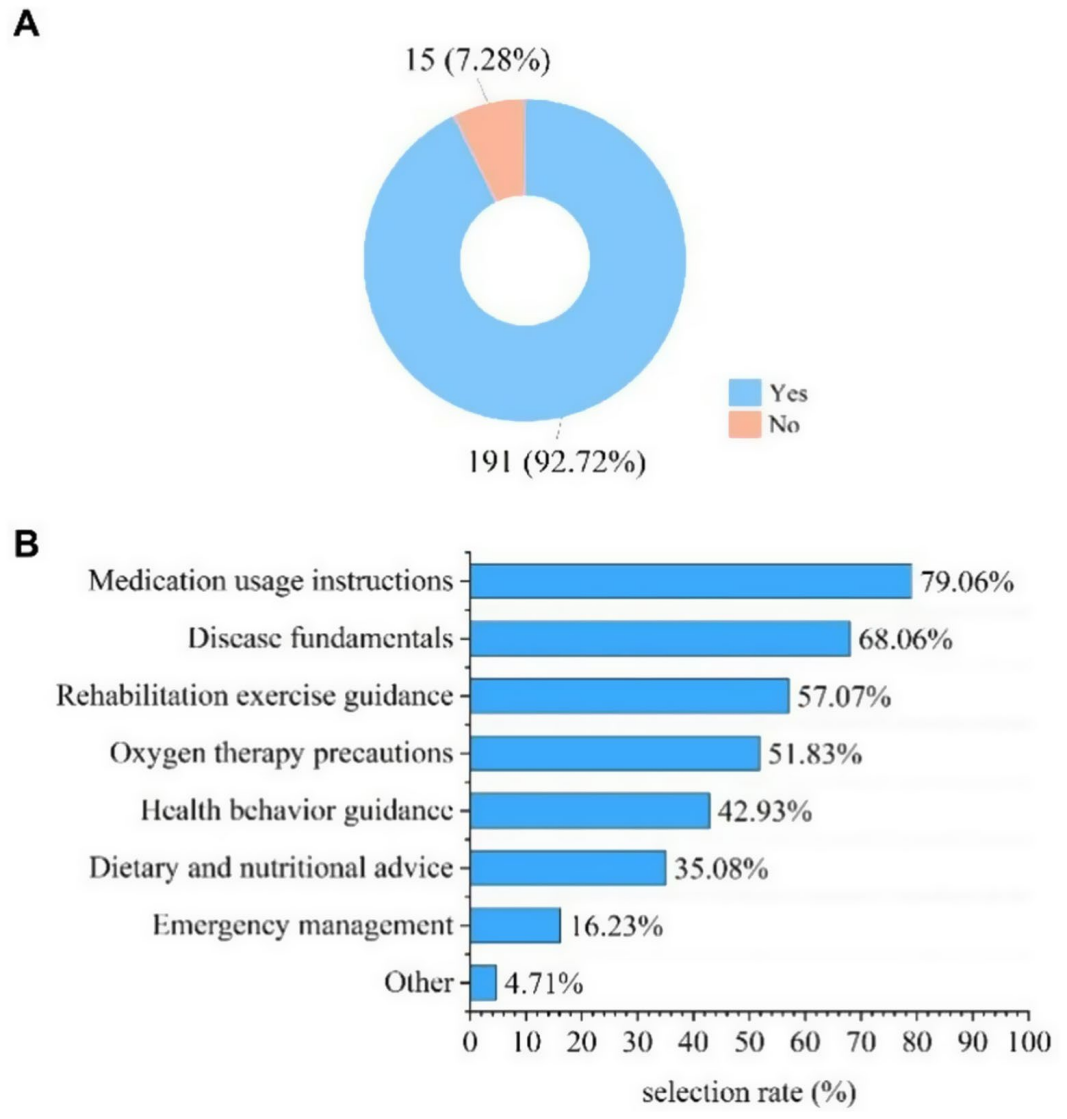
Health education received by patients. **(A)** Receipt of PH-related health education (*n* = 206). **(B)** Types of health education content received (multiple responses allowed; *n* = 191).

**Figure 2:**
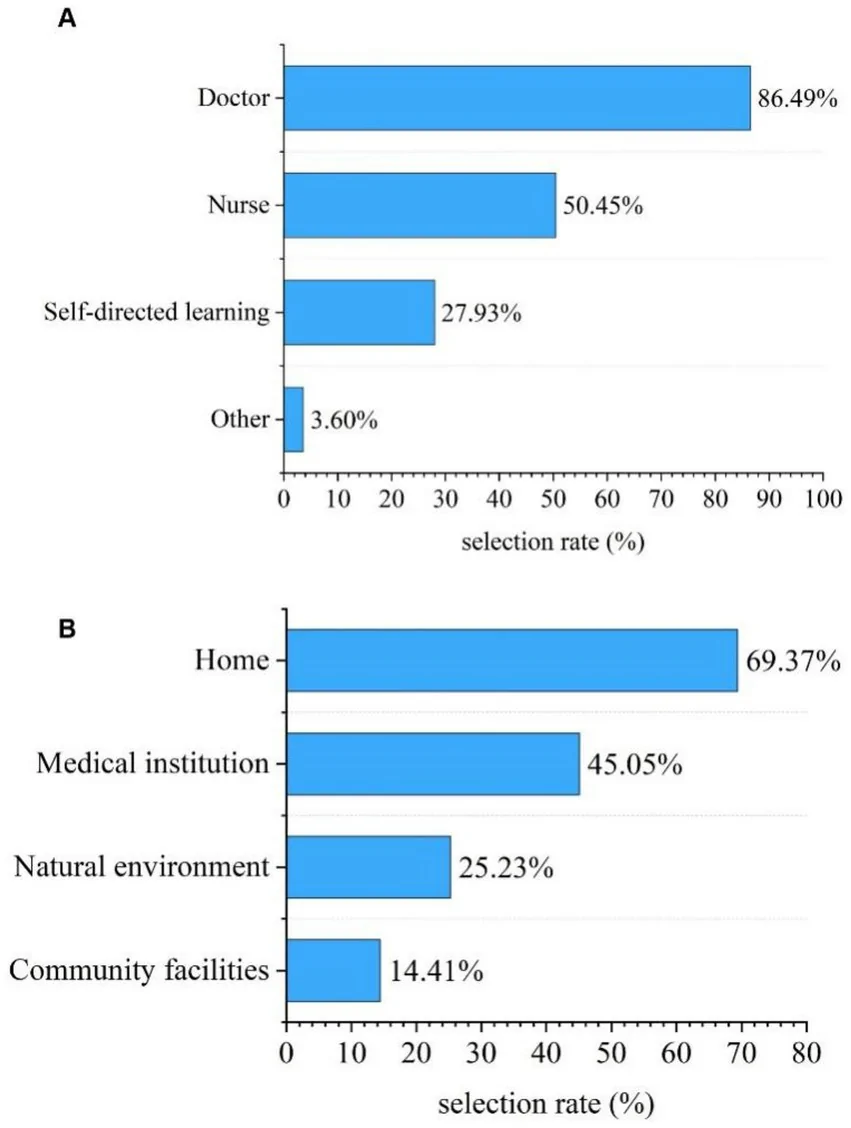
Implementation characteristics of PR exercises (multiple responses allowed; *n* = 111). **(A)** Sources of rehabilitation guidance. **(B)** Primary locations where patients conduct rehabilitation training.

A total of 92.72% of patients reported that they had received health education, focusing primarily on medication usage instructions, disease fundamentals, and rehabilitation exercise guidance (Figure 1). Overall, 53.88% of participants reported engaging in breathing exercises or physical training, with 58.56% reporting a frequency of 1–2 times daily. Regarding family assistance, 69.37% reported that family members assisted with rehabilitation training, while 25.23% reported occasional assistance. 90.09% reported improved dyspnea after exercise, and 87.39% reported improved fatigue after exercise. Detailed results are shown in Table 2. These rehabilitation activities were primarily guided by medical staff, and most were performed at home (Figure 2).

**Table 2:** Current status of PR.

Item	Frequency (*n*)	Percentage (%)	Cumulative percentage (%)
Whether you have engaged breathing exercises or physical training? (*n* = 206)			
Yes	111	53.88	53.88
No	95	46.12	100.00
How often do you perform rehabilitation training? (*n* = 111)			
1–2 times daily	65	58.56	58.56
3–5 times weekly	14	12.61	71.17
1–2 times weekly	8	7.21	78.38
Irregularly	24	21.62	100.00
Whether family members assist you with rehabilitation training? (*n* = 111)			
Yes	77	69.37	69.37
Occasionally	28	25.23	94.59
No	6	5.41	100.00
Whether your dyspnea symptoms have improved after exercise? (*n* = 111)			
Significantly improved	49	44.14	44.14
Slightly improved	51	45.95	90.09
No change	9	8.11	98.20
Worsened	2	1.80	100.00
Whether your fatigue symptoms have improved after exercise? (*n* = 111)			
Significantly improved	37	33.33	33.33
Slightly improved	60	54.05	87.39
No change	12	10.81	98.20
Worsened	2	1.80	100.00

#### 3.1.3 Rehabilitation barriers and support needs

**Figure 3:**
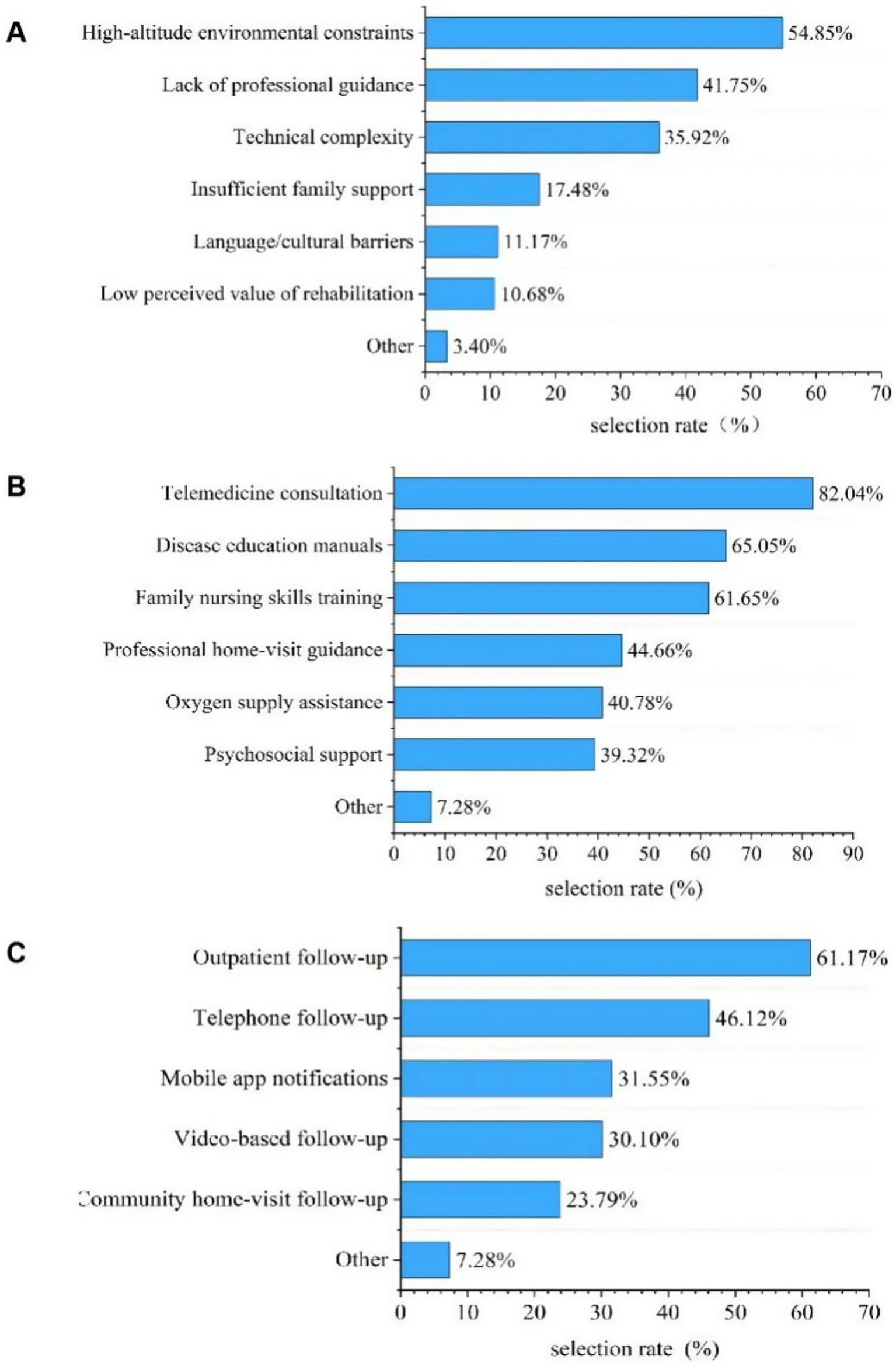
Patient-reported barriers to PR and support needs (multiple responses allowed; *n* = 206). **(A)** Factors affecting participation in rehabilitation exercises. **(B)** Types of rehabilitation support desired by patients. **(C)** Preferred follow-up methods after discharge.

Participants identified the main barriers affecting PR as high-altitude environmental constraints (54.85%), lack of professional guidance (41.75%), and technical complexity such as difficulty in mastering breathing training techniques (35.92%). The top three desired rehabilitation supports were telemedicine consultation (82.04%), disease educational materials (65.05%), and family nursing skills training (61.65%). The top three preferred follow-up methods were outpatient follow-up (61.17%), telephone follow-up (46.12%), and mobile app notification (31.55%). Detailed results are shown in Figure 3.

#### 3.1.4 Additional suggestions

No participants responded to the open-ended questionnaire items.

### 3.2 Qualitative research results

A total of 11 patients with PH were interviewed. The cohort comprised 7 males and 4 females, 10 Han Chinese and 1 Tibetan, aged between 59 and 82 years, with a disease duration of 3 to 15 years. All residents lived in areas above 2,000 m in altitude (Table 3). Through thematic analysis, three core themes and eight subthemes were extracted.

**Table 3:** Baseline characteristics of interviewed patients.

Participants	Sex	Age (years)	Ethnicity	Occupation	Average altitude of residence (m)	Disease duration (years)
1	Male	78	Han	Retired	2,231	3
2	Female	69	Han	Unemployed	2,231	5
3	Male	63	Han	Farmer	2,520	10
4	Male	75	Han	Farmer	2,300	10
5	Male	82	Han	Retired	2,300	10
6	Male	62	Tibetan	Farmer	3,300	5
7	Female	76	Han	Unemployed	2,231	6
8	Female	59	Han	Unemployed	2,520	10
9	Female	60	Han	Farmer	2,780	15
10	Male	64	Han	Farmer	2,520	10
11	Male	73	Han	Farmer	2,050	4

#### 3.2.1 Theme 1: current PR practices and perceived symptom changes

In-hospital PR measures: Some interviewees reported not receiving guidance on PR during hospitalization, stating: “I have not received guidance on these PR measures” (Participant 2), “No guidance was provided” (Participant 4), and “No, basically, it was just routine examinations and treatment according to their procedures” (Participant 6). In contrast, interviewees who received guidance noted that during hospitalization, the hospital organized activities such as Baduanjin exercises, breathing training, and pulmonary function exercises using tools (e.g., blowing up balloons). They were also advised to walk more, supplemented by oxygen therapy.

Home-based PR measures: Interviewees indicated that walking was their primary form of exercise at home, with other activities including practicing tai chi and tai chi sword, dancing Guozhuang (a traditional Tibetan folk dance), square dancing, and home oxygen therapy.

Perceived symptom changes: Interviewees reported perceived improvements in their symptoms after engaging in PR activities. Verbatim supporting quotations for these subthemes are provided in Table 4.

**Table 4:** Current PR practices and perceived symptom changes.

Subtheme	Illustrative
In-hospital pulmonary rehabilitation measures	“Baduanjin, they play the video over the radio. Lots of people do it at the hospital. As soon as the radio starts, they shout: ‘Patients, come out and do the exercises!’ At Huzhu County Hospital, it only lasts a little while, no more than five minutes at most. I have not done Baduanjin at home, I almost only do it at the hospital.” (Participant 1) “Just inhale through the nose and exhale through the mouth. I do it two or three times a day, taking five or six breaths each time. They told me to blow up balloons, I blow for a while at noon and a while in the evening, about two or three minutes each time.” (Participant 8) “The doctor said to exercise moderately, just walking, and also oxygen therapy. I do not walk much every day, just a few hundred meters. I use oxygen every day, almost all the time except when I’m active.” (Participant 11)
Home-based pulmonary rehabilitation measures	“At home, I do tai chi and tai chi sword pretty well, and sometimes I dance Guozhuang. I’m Han nationality, so I still prefer my tai chi and tai chi sword. When I feel tired, I do my tai chi and tai chi sword.” (Participant 1) “At home, I dance square dancing and such. At first, I did tai chi and tai chi exercises, then switched to square dancing, doing it every day.” (Participant 2) “Oxygen therapy, I basically do it once a day, for about an hour and a half to two hours. I use it when I feel uncomfortable.” (Participant 3) “For my own exercise, I just walk every day. About two hours a day, on average.” (Participant 5) “I have oxygen canisters. I usually use oxygen for one to two hours before bed every day. Because back then, I probably had low oxygen, so we go to the hospital to refill the oxygen ourselves.” (Participant 6)
Perceived symptom changes	“It’s improved a lot, the effect is quite significant. I’m nearly 80 now, and I rely on tai chi and tai chi sword.” (Participant 1) “Yes, there’s a slight effect.” (Participant 5) “Yes, when I cannot catch my breath, blowing up balloons four or five times makes me feel better.” (Participant 8) “It’s just that I feel short of breath, but after using oxygen, it slowly gets better.” (Participant 11)

#### 3.2.2 Theme 2: barriers to PR implementation

Physical conditions: Interviewees generally reported that somatic symptoms such as dyspnea, persistent fatigue, and chest tightness significantly reduced their willingness and ability to persist in rehabilitation exercises, serving as direct physiological barriers to daily exercise.

Insufficient awareness of PR: Most interviewees clearly stated that they lacked a basic understanding of the concept of PR, including its specific connotations, operational methods, and potential benefits. This cognitive gap further restricted their initiative to actively engage in rehabilitation. Verbatim supporting quotations for these subthemes are provided in Table 5.

**Table 5:** Barriers to PR implementation.

Subtheme	Illustrative
Physical conditions:	“After getting sick, I stopped dancing. I cannot catch my breath, and after exercising a little, I get extremely tired.” (Participant 2) “I cannot keep it up. Even if I try to do it, I cannot last long. After doing it two or three times, I get short of breath.” (Participant 7) “I cannot walk too far or too fast. After walking for a while, my chest feels uncomfortable and I cannot catch my breath.” (Participant 9)
Insufficient awareness of PR	“Actually, most people have absolutely no idea what pulmonary rehabilitation is. They do not know what ‘rehabilitation’ means. So when you talk about pulmonary rehabilitation, a lot of people do not know what that means. I have many friends in the medical circle who run businesses in rehabilitation, and from what I know, people did not have this concept before at all, they did not even know what rehabilitation was. It’s only in the past few years that this work has started to take off.” (Participant 5) “We do not really understand what pulmonary rehabilitation is either, so we cannot offer any such suggestions.” (Participant 6)

#### 3.2.3 Theme 3: support needs related to PR

Enhancing post-discharge health education: Interviewees emphasized that current health education is inadequate and expressed a strong desire for systematic and clear guidance at discharge, including daily precautions, self-care tips, and medication advice.

Community-provided instructional activities: Interviewees suggested that hospital experts with rehabilitation experience could visit communities to offer professional guidance. Communities could also organize regular rehabilitation-related activities or provide personalized guidance through home visits.

Leveraging digital health platforms: Interviewees expressed a need for support in home PR exercises through internet platforms (e.g., short videos, official accounts, WeChat groups) or phone calls, allowing them to conveniently practice at home. Verbatim supporting quotations for these subthemes are provided in Table 6.

**Table 6:** Support needs related to PR.

Subtheme	Illustrative
Enhancing post-discharge health education post-discharge health education	“Right now, once you are discharged from the hospital, there’s no follow-up at all. We hope that when we are discharged each time, they’ll clearly tell us what to pay attention to in daily life, which aspects to focus on for self-care at home, what we should note after discharge, or what we should do ourselves when we encounter such situations. Things like which medicines work better to take, and what we can do on our own when symptoms are mild, these kinds of things.” (Participant 3) “When we were seeing doctors before, the doctor said to go to low-altitude, high-oxygen areas. Because we live in that pastoral area, Gangcha County has a high altitude. But basically, once you are discharged, there’s no follow-up care.” (Participant 6)
Community-provided instructional activities	“The best thing would be for hospital experts with rehabilitation experience to go down to communities to provide guidance. Also, communities should take the lead, then organize the older adults and arrange activities based on what they enjoy doing.” (Participant 1) “Like, if communities have our names and information, they could come over to check on us, ask how we are doing, and provide door-to-door guidance.” (Participant 8) “It would definitely be good if communities could take time to provide guidance in this area.” (Participant 11)
Leveraging digital health platforms	“Things like Douyin (the Chinese version of TikTok), short videos, or official WeChat accounts, we could follow them ourselves and learn at home by following along. WeChat groups would also work. If there were such resources, we would follow them, checking in every day to learn.” (Participant 3) “They could push videos through WeChat or these platforms.” (Participant 5) “If they could make regular follow-up calls to check in, and we could share any issues we have, that would work.” (Participant 11)

### 3.3 Integrated interpretation of results

The quantitative and qualitative findings were largely complementary while also revealing several areas of divergence.

Both datasets suggested that participants perceived improvements. On current practice, the quantitative data showed that just over half of patients (53.88%) performed PR exercises, while qualitative interviews filled in the details by describing exactly what those exercises looked like (Baduanjin, walking, tai chi) and where they happened (hospital wards, home, community spaces). Both datasets indicated that participants perceived improvements in dyspnea and fatigue after engaging in PR activities.

On barriers, the quantitative survey identified environmental constraints and lack of professional guidance as the leading obstacles. The qualitative interviews added contextual insights by highlighting physical symptoms and limited awareness of PR as important barriers to regular participation.

On support needs, the two methods converged strongly. The high demand for telemedicine (82.04%) matched qualitative calls for online guidance; the desire for educational materials and family caregiver training aligned with requests for structured discharge teaching and community-based activities.

## 4 Discussion

This study provides a patient-centered examination of PR practices for PH in high-altitude areas using a convergent parallel mixed-methods design to quantify gaps and explore the underlying reasons. By integrating quantitative survey findings with qualitative interview data, we obtained a comprehensive understanding of patients’ PR practices, barriers, and support needs. This approach enabled us to identify not only the extent of PR participation but also the underlying barriers and patients’ perceived support needs. Because this study focused primarily on patient-reported rehabilitation experiences, our findings reflect perceived outcomes rather than differences attributable to disease severity.

### 4.1 Current PR practice: partial uptake with uneven quality

A fundamental finding from the quantitative survey was that over half (53.88%) of the surveyed PH patients had engaged in some form of PR exercise, suggesting that PR had been implemented to some extent among this patient population. However, this overall participation rate masked substantial disparities in the quality and accessibility of PR guidance, particularly during hospitalization. Qualitative interviews revealed a stark contrast: some patients received structured inpatient PR activities, including Baduanjin (a traditional Chinese qigong practice), breathing training, tool-assisted pulmonary function exercises (e.g., balloon blowing), and personalized advice to increase walking combined with oxygen therapy, while others received no formal PR guidance at all. The apparent inconsistency between the quantitative and qualitative findings may reflect differences in patients’ perceptions of the adequacy and quality of PR guidance rather than its mere provision. Although most participants reported receiving health education, rehabilitation exercise guidance represented only one component of the educational content, and receipt of health education did not necessarily translate into participation in PR exercise.

The lack of standardization is not unique to high-altitude settings; similar inconsistencies have been documented even in lowland areas (16, 17). However, the consequences are magnified here: patients who leave the hospital without proper guidance may be less prepared to perform home-based PR safely and effectively, while those receiving structured guidance may be better prepared to continue exercising after discharge.

Medical institutions often face resource constraints, including a shortage of professional rehabilitation personnel, insufficient training of general medical staff in PR, and conflicts in prioritization between managing acute exacerbations of PH and providing preventive rehabilitation (18, 19). Patients who do not receive in-hospital guidance may lack the basic knowledge required to carry out safe and effective home-based PR, increasing the risk of exercise-induced symptom exacerbation or complete withdrawal from rehabilitation. This issue is compounded by unique challenges in high-altitude areas.

Home-based PR described by interviewees reflects a combination of necessity and cultural adaptability. Walking emerged as the primary activity due to its accessibility and low cost, which are crucial factors in resource-limited settings (20). Beyond walking, patients also incorporated culturally relevant activities such as tai chi, tai chi sword, and square dancing. The integration of cultural activities into PR is noteworthy; for example, square dancing not only provides physical activity but also promotes social interaction and strengthens community support (21, 22). However, the lack of professional supervision in home settings raises concerns about exercise intensity, duration, and safety. Without guidance, patients may either under-exercise due to fear of worsening symptoms or over-exercise, facing the risk of hypoxia-related complications in high-altitude environments (23). This underscores the necessity of bridging inpatient and home-based PR through personalized exercise plans and follow-up checks.

### 4.2 Patient-perceived symptom changes following PR participation

Both quantitative and qualitative findings consistently suggested that participants perceived symptom benefits after engaging in PR activities. The quantitative data showed that 90.09% of participants reported improvement in dyspnea and 87.39% reported improvement in fatigue after exercise. In qualitative interviews, participants also provided feedback on similar improvements. These patient-reported findings are broadly consistent with previous studies suggesting potential benefits of PR in patients with PH.

The mechanisms underlying these improvements warrant in-depth exploration. Exercise-based PR, represented by walking and structured breathing training, enhances cardiopulmonary efficiency by strengthening respiratory muscle strength, improving oxygen utilization, and reducing ventilatory demand (24). Such adaptive changes are particularly crucial in high-altitude environments characterized by reduced ambient oxygen availability. For instance, breathing training taught in hospitals may help optimize gas exchange and alleviate hypoxic stress. Traditional activities such as Baduanjin, tai chi, square dancing, and Guozhuang dance may also support rehabilitation. Their coordinated breathing, gentle movements, and culturally familiar nature may improve respiratory function and exercise tolerance while encouraging participation and long-term adherence (25, 26). The quantitative findings describe the proportion of participants who perceived symptom improvement, while the qualitative narratives provide contextual insight into how participants experienced these changes in daily life. This convergence suggests that participants recognized potential benefits of PR and highlights the need for further prospective evaluation in this population. However, whether different PR modalities are associated with different clinical outcomes remains unclear and should be examined in future prospective intervention studies to help refine PR recommendations.

### 4.3 Barriers: a tangle of environment, knowledge, and resources

The identification of barriers to PR implementation reveals a complex interplay of environmental, physical, and systemic factors. Quantitative data indicate that high-altitude environmental constraints are the primary barrier, followed by a lack of professional guidance and technical complexity. Qualitative data further expand on these findings, emphasizing that physical limitations (dyspnea, fatigue, chest tightness) and insufficient awareness of PR are core sub-themes. Although environmental constraints emerged as the most frequently reported barrier in the quantitative survey, they were less prominent in the qualitative interviews. One possible explanation is that most participants had lived at high altitude for many years and regarded the hypoxic environment as an unavoidable part of everyday life rather than a distinct rehabilitation barrier. Consequently, during interviews they tended to focus on barriers they perceived as potentially modifiable, such as physical symptoms, insufficient knowledge of PR, and lack of professional guidance. This apparent difference between the quantitative and qualitative findings does not necessarily indicate inconsistency; rather, it reflects the different ways in which structured questionnaires and open-ended interviews capture participants’ experiences. Together, these findings outline the unique obstacles faced by this population, which urgently require targeted solutions.

High-altitude environments exert a profound impact on the feasibility of PR. A total of 88.83% of participants reside at elevations above 2000 meters, where hypobaric hypoxia reduces maximal oxygen uptake, limits exercise capacity, and increases the perceived exertion of even mild activities (27). Cold temperatures, harsh weather, and rugged terrain further restrict outdoor exercise (28), which is the primary form of PR for many patients. These environmental factors interact with physical symptoms: dyspnea and fatigue, hallmarks of PH, create a vicious cycle in which exercise-induced discomfort reduces motivation, leading to deconditioning and further symptom exacerbation (29, 30). For patients with severe PH, breaking this cycle is particularly challenging without structured support. Therefore, to address the high-altitude environmental and physical limitations, indoor PR facilities and personalized exercise plans may be considered, incorporating remote monitoring to mitigate environmental impact and provide structured support to break the cycle of symptoms.

The lack of professional guidance, identified in both quantitative and qualitative data, stems from multiple causes. First, high-altitude areas often suffer from a shortage of healthcare providers trained in PR. Many institutions prioritize acute care over preventive rehabilitation, leaving limited time and resources for patient education (31). Second, patients have insufficient awareness of PR. Qualitative interviewees expressed confusion about what PR entails, including its goals, methods, and benefits. This knowledge gap is exacerbated by inadequate health education (32). Therefore, training local healthcare professionals, integrating PR into primary healthcare, and launching community health education programs could be beneficial strategies to enhance patients’ understanding and participation.

Cultural and socioeconomic factors also play a role. The study population includes eight ethnic groups, with cultural practices shaping health behaviors. While traditional activities like tai chi offer opportunities for culturally congruent PR, they may lack integration with formal medical recommendations, limiting their effectiveness. Additionally, 23.30% of patients reported that disease treatment costs severely impact their daily lives, potentially restricting access to PR tools, transportation to medical facilities, or participation in paid rehabilitation programs. These socioeconomic barriers further widen disparities in PR implementation rates (33, 34). Additionally, traditional cultural activities could be incorporated into standardized PR protocols, and financial subsidies or insurance coverage may help alleviate cost burdens and improve accessibility.

### 4.4 Support needs: building a patient-centered support system

Patient-reported support needs, identified in both quantitative and qualitative data, provide a roadmap for improving PR services in high-altitude areas. Quantitative data show that the most desired forms of support are telemedicine consultations, disease knowledge popularization manuals, and family care skills training. From a qualitative perspective, patients emphasized strengthened discharge education, community activities, and remote home guidance. These needs reflect a clear desire for accessible, personalized support to address geographic isolation and resource limitations. There is a strong demand for accessible support, including telemedicine, educational resources, and community-based programs.

Telemedicine and digital health solutions have become key tools for overcoming geographical barriers. In high-altitude areas where 33.50% of patients spend more than 1 h on a one-way trip to the nearest medical institution, remote guidance can bridge the gap in access to professional services. Within this framework of addressing geographic challenges, patients’ preferences for follow-up methods also deserve attention. Outpatient follow-up remains their top choice for post-treatment monitoring, as it enables face-to-face assessments by specialists, comprehensive physical examinations, and direct adjustments to rehabilitation plans based on real-time progress. This in-person interaction provides patients with a greater sense of security, especially when addressing complex symptoms or concerns requiring hands-on evaluation (35, 36). Meanwhile, telephone follow-ups, preferred by 46.12% of patients, offer a low-tech alternative for populations with limited digital access, ensuring coverage across all age and socioeconomic groups. Studies have confirmed that telemedicine improves PR adherence in patients through real-time feedback, exercise monitoring, and symptom classification, with such benefits being more pronounced in underserved areas (37, 38). Mobile applications or video platforms can further deliver culturally tailored content, ensuring recommendations align with local practices.

Community support is equally crucial. Patients suggested that regular PR activities be organized locally. Such a model could leverage existing social structures, such as community centers or cultural groups, to deliver group-based rehabilitation while fostering social support and improving access to rehabilitation services (39). Community health workers trained in basic PR principles can play a crucial role in maintaining activities, conducting home visits, and monitoring patient progress. This approach may improve accessibility and facilitate the integration of PR into daily life. These findings support the further development and evaluation of context-specific PR strategies tailored to high-altitude settings.

Enhanced discharge education addresses the transition gap between hospital and home. Patients have reported insufficient current education, highlighting the need for structured discharge plans that include personalized exercise prescriptions, symptom management strategies, and clear follow-up protocols. Educational materials need to be culturally and linguistically appropriate, with visual aids or verbal instructions provided for patients with low literacy levels (40). By equipping patients with knowledge and skills at discharge, healthcare providers can empower them to safely and effectively self-manage PR, which may support safer self-management and potentially reduce rehospitalization risk. These efforts may contribute to improving long-term functional outcomes and quality of life in this vulnerable population.

No participants provided responses to the open-ended questionnaire items. This may reflect questionnaire fatigue after completing the structured items or a preference for responding to closed-ended questions in an online survey. Nevertheless, the subsequent qualitative interviews enabled participants to provide more detailed accounts of their experiences and support needs, thereby complementing the absence of open-ended questionnaire responses.

Our previous study explored PR from the perspective of medical staff (12), whereas the present study complements those findings by incorporating patients’ experiences and support needs. Together, these complementary findings provide a more comprehensive understanding of PR implementation in high-altitude areas and support the development of patient-centered rehabilitation strategies.

### 4.5 Strengths and limitations

The mixed-methods design is a major strength of this study. The quantitative survey quantified PR participation, barriers, and support needs, while the qualitative interviews provided contextual explanations for these findings. The integration of both datasets offered a more comprehensive understanding of PR implementation from the patient perspective. Several limitations should be acknowledged. First, this was a single-center study using convenience sampling, which may limit the generalizability of the findings to other high-altitude regions or healthcare settings. Second, although family caregivers were permitted to assist eligible patients in completing the online questionnaire, recruitment through a QR-code-based survey at hospital discharge may still have introduced selection bias by underrepresenting patients or caregivers unable or unwilling to use digital platforms. The online platform did not distinguish between questionnaires completed independently by patients and those completed with caregiver assistance, preventing assessment of potential proxy-response bias. Third, the qualitative sample was relatively small and recruited from questionnaire respondents who were willing and able to participate in interviews. In addition, the qualitative interview sample consisted predominantly of Han Chinese participants. Therefore, the diversity of perspectives may have been limited despite achieving data saturation, and the transferability of the qualitative findings to other ethnic groups living in high-altitude areas should be interpreted with caution. Fourth, detailed clinical characteristics, including WHO functional class, baseline SpO_2_, and 6-min walk distance, were not collected as part of the study, precluding analyses according to disease subtype or severity. Finally, cultural factors, such as language differences, health literacy, and traditional health beliefs, were not explored in depth. Future studies should evaluate the implementation and effectiveness of context-specific PR interventions developed based on these findings.

## 5 Conclusion

This study found that many patients perceived improvements in dyspnea and fatigue after engaging in PR activities, although implementation remained variable and uneven. Environmental constraints, insufficient professional guidance, and limited patient awareness remained major barriers. Patients expressed strong preferences for telemedicine, educational materials, community-based programs, and improved discharge planning. These findings also highlight the need to strengthen standardized inpatient PR education and structured discharge planning to ensure continuity of rehabilitation from hospital to home. Together with existing evidence, our findings may help inform the development of context-specific PR programs and digital rehabilitation support strategies for patients with PH living at high altitude.

## Data Availability

The raw data supporting the conclusions of this article will be made available by the authors, without undue reservation.
