# Supplementary material for: Patients’ perspectives on pulmonary rehabilitation for pulmonary hypertension in high-altitude areas: a mixed-methods study
Source: Front Public Health. 2026 Aug 17;14:1869309. doi: 10.3389/fpubh.2026.1869309 (PMC13526976; doi:10.3389/fpubh.2026.1869309)
Supplement: Supplementary file 1 [file Data_Sheet_1.DOCX]

**Supplementary Material**

Questionnaire on the Pulmonary Rehabilitation Status of Patients with Pulmonary Hypertension in High-Altitude Areas (*For Patients*)

Dear patient friends,

Hello! We are currently conducting a survey on the pulmonary rehabilitation status for patients with pulmonary hypertension in high-altitude areas. This study aims to understand the pulmonary rehabilitation current practices, barriers, and support needs in the unique high-altitude environment, so as to provide a basis for developing more scientific and comprehensive pulmonary rehabilitation plans.

Your participation is highly important! This survey is anonymous and will not disclose any of your personal information. All data will be used solely for academic research. The questionnaire will take approximately 8-10 minutes to complete. Your valuable feedback will directly contribute to improving pulmonary rehabilitation services for pulmonary hypertension patients in high-altitude areas, benefiting more patients. We sincerely appreciate your support and cooperation!

Friendly reminder: Complete the survey to receive a cash reward. Click below “I agree” to start filling it out ↓

1. What is your biological sex?

Male

Female

2. Which age group do you belong to?

18–25 years

26–30 years

31–40 years

41–50 years

51–60 years

≥61 years

3. What is your ethnicity? (Options were provided through a dropdown menu on the “Wenjuanxing” platform, allowing patients to select their ethnic group)
4. What is your current employment status?

Manual laborer

Casual/part-time worker

Retired

Student

Unemployed

Homemaker

Professional/manager

Other

5. Where is your household located? (To avoid potential uncertainty regarding self-reported altitude data, we had opted to collect patients' residential addresses instead. On the “Wenjuanxing” platform, respondents selected their province from a dropdown menu, which then populated all available cities within that province. After selecting a city, all corresponding counties/districts were displayed. Following the collection of the questionnaires, we categorized the altitude data by cross-referencing each respondent's provided location with official geographical records)
6. How long you have lived at your current location?

1–5 years

6–10 years

11–20 years

21–30 years

≥31 years

7. What is your current smoking status?

Never smoked

Current smoker

Former smoker

8. What is your current drinking status?

Never drank

Current drinker

Former drinker

9. How many times were you hospitalized for pulmonary hypertension in the past year (excluding the current hospitalization)?

0 times

1 time

2 times

3 times

4 times

≥5 times

10. Whether your household has home oxygen therapy equipment?

Yes

No

11. How long does it take you to travel to the nearest medical facility (one-way)?

< 30 minutes

30–60 minutes

1–2 hours

> 2 hours

12. How does the cost of your treatment impact your daily life?

No impact

Some impact

Severe impact

13. How many family members live with you?

None

1 person

2 persons

3 persons

≥4 persons

14. Who is your primary caregiver?

Children

Spouse

Other

15. Whether you have received health education related to pulmonary hypertension? (Select "yes" to continue to the next question, or select "no" to skip to Question 17.)

Yes

No

16. What health education content have you received? (Multiple choices)

disease fundamentals

Medication usage instructions

Rehabilitation exercise guidance

Dietary and nutritional advice

Disease fundamentals

Health behavior guidance

Emergency management

Oxygen therapy precautions

Other

17. Whether you have engaged breathing exercises or physical training? (Select "yes" to continue to the next question, or select "no" to skip to Question 24.)

Yes

No

18. How often do you perform rehabilitation training?

1–2 times daily

3–5 times weekly

1–2 times weekly

Irregularly

19. Who guided the rehabilitation training for you? (Multiple choices)

Doctor

Nurse

Self-directed learning

Other

20. Where do you primarily conduct rehabilitation training? (Multiple choices)

Medical institution

Home

Natural environment

Community facilities

Other

21. Whether family members assist you with rehabilitation training?

Yes

No

Occasionally

22. Whether your dyspnea symptoms have improved after exercise?

Significantly improvement

Slightly improvement

No change

Worsened

23. Whether your fatigue symptoms have improved after exercise?

Significantly improvement

Slightly improvement

No change

Worsened

24. What factors affect your participation in rehabilitation exercises? (Multiple choices)

Language/cultural barriers

High-altitude environmental constraints

Lack of professional guidance

Technical complexity (such as difficulty in mastering breathing training techniques)

Insufficient family support

Low perceived value of rehabilitation

Other

25. What types of rehabilitation support do you hope to receive? (Multiple choices)

Disease education manuals

Family nursing skills training

Psychosocial support

Telemedicine consultation

Oxygen supply assistance

Professional home-visit guidance

Other

26. Which follow-up methods do you prefer for reexamination or condition updates? (Multiple choices)

Mobile app notifications (after receiving the reminder, you filled in the rehabilitation status online)

Telephone follow-up

Outpatient follow-up

Community home-visit follow-up

Video-based follow-up

Other

27. Do you have any other opinions or suggestions regarding pulmonary arterial hypertension rehabilitation services? (Optional)

**Supplementary Material**

Table S1 Interview guide.

| Number | Content |
| --- | --- |
| 1 | Which pulmonary rehabilitation methods are you currently undertaking, and what is their frequency? Who supervises or guides these measures? |
| 2 | Have these rehabilitation measures led to any improvement in your symptoms? |
| 3 | What is the most significant difficulty or barrier you encounter in consistently adhering to these rehabilitation measures? |
| 4 | Beyond the pulmonary rehabilitation methods you are currently using, in which other areas related to your condition or well-being would you like to receive further rehabilitation guidance or support? |
| 5 | What type of setting would you prefer to receive future pulmonary rehabilitation treatment? |
| 6 | Which specific technological tools, devices, or applications do you believe could potentially enhance the effectiveness or your adherence to pulmonary rehabilitation? |
| 7 | Do you have any additional suggestions regarding the pulmonary rehabilitation services? |
